# Supplementary figures and images for: Evaluating the longitudinal physical and psychological health effects of persistent long Covid 3.5 years after infection
Source: PLoS One. 2025 Jun 24;20(6):e0326790. doi: 10.1371/journal.pone.0326790 (PMC12186912; doi:10.1371/journal.pone.0326790)

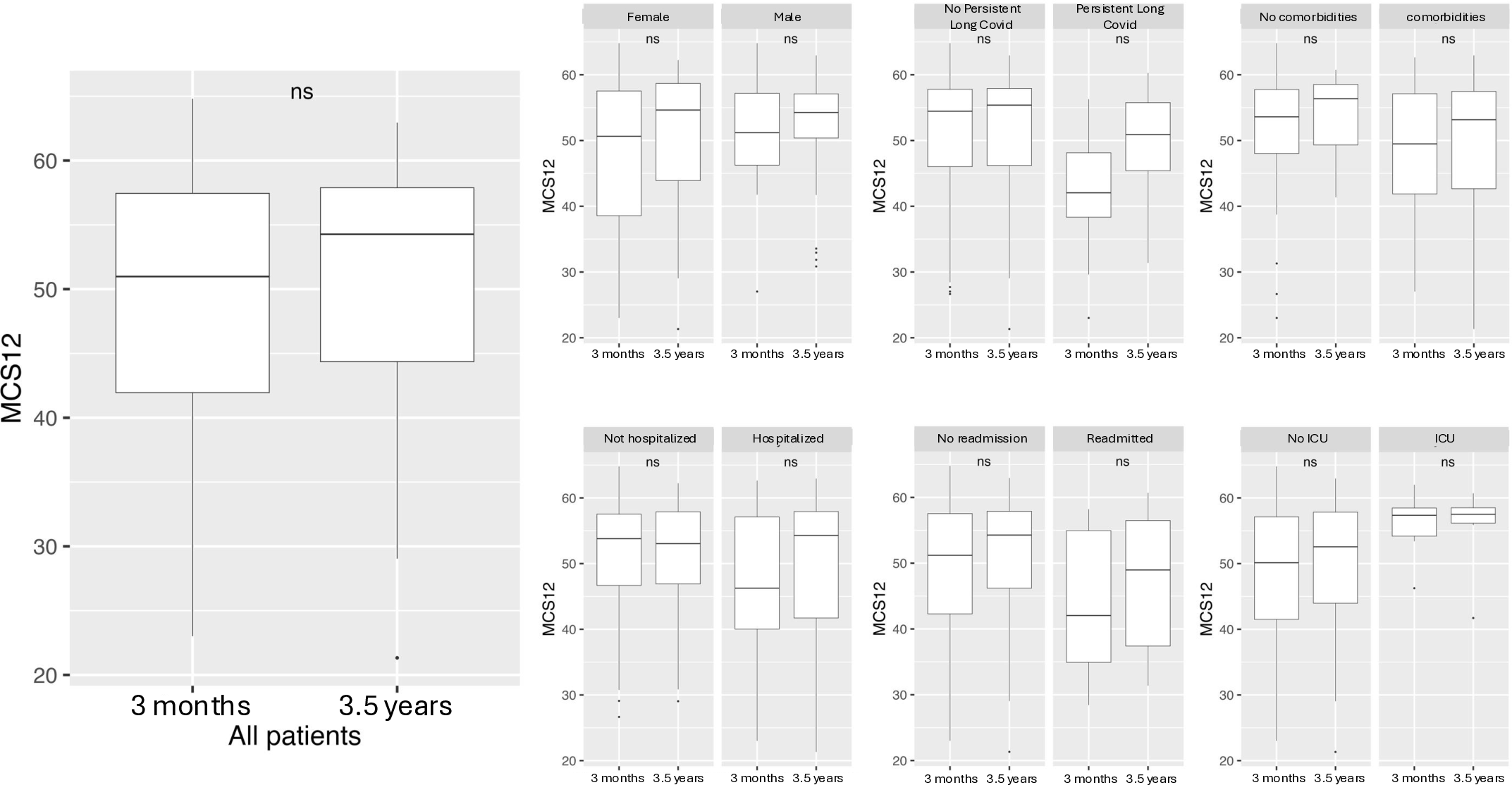

Supplement: S1 Fig — Comparison of all patients. Subgroups analyses comparing patients by sex, persistent Long Covid status, Presence of comorbidities, hospitalization status, hospital readmissions and ICU care requirement on admission. (TIF) [file pone.0326790.s001.tif]
