# Supplementary material for: Evaluating the longitudinal physical and psychological health effects of persistent long Covid 3.5 years after infection
Source: PLoS One. 2025 Jun 24;20(6):e0326790. doi: 10.1371/journal.pone.0326790 (PMC12186912; doi:10.1371/journal.pone.0326790)
Supplement: S1 Table — N = number of Patients. CI = Confidence interval. (PDF) [file pone.0326790.s002.pdf]

Acute symptoms at initial presentation	3 month		1 year		3.5 years	
	N (%)	95%CI	N (%)	95%CI	N (%)	95%CI
Total patients with symptom information	142		89		83	
Shortness of breath	69 (49%)	41-57%	46 (52%)	42-62%	43 (52%)	41-63%
Cough	79 (56%)	48-64%	50 (56%)	46-66%	41 (49%)	38-60%
Fever	66 (46%)	38-54%	42 (47%)	37-57%	36 (43%)	32-54%
Myalgia	57 (40%)	32-48%	33 (37%)	27-47%	32 (39%)	29-49%
Headache	42 (30%)	23-38%	26 (29%)	20-38%	23 (28%)	18-38%
Anosmia	39 (27%)	20-34%	22 (25%)	16-34%	22 (27%)	18-37%
Chest pain	22 (15%)	9-21%	15 (17%)	9-25%	13 (16%)	8-24%
Sore Throat	23 (16%)	10-22%	16 (18%)	10-26%	11 (13%)	6-20%
Nausea/Vomiting	11 (8%)	4-12%	6 (7%)	2-12%	6 (7%)	2-13%
Diarrhoea	11 (8%)	4-12%	6 (7%)	2-12%	6 (7%)	2-13%
Joint pain	4 (3%)	0-6%	3 (3%)	0-7%	3 (4%)	0-8%
Pneumonia	3 (2%)	0-4%	2 (2%)	0-5%	3 (4%)	0-8%
Runny nose	6 (4%)	1-7%	1 (1%)	0-3%	2 (2%)	0-5%
Abdominal pain	4 (3%)	0-6%	3 (3%)	0-7%	2 (2%)	0-5%
Weakness	3 (2%)	0-4%	2 (2%)	0-5%	2 (2%)	0-5%
