# Supplementary material for: Evaluating the longitudinal physical and psychological health effects of persistent long Covid 3.5 years after infection
Source: PLoS One. 2025 Jun 24;20(6):e0326790. doi: 10.1371/journal.pone.0326790 (PMC12186912; doi:10.1371/journal.pone.0326790)
Supplement: S2 Table — A Test was conducted on the difference between means of the paired data at the respective timepoints. (PDF) [file pone.0326790.s003.pdf]

SF-12 subscores	Timepoint comparison	Population	subgroup	Shapiro-wilk test[a]
PCS12	3M vs 4 year	total group		W = 0.98867, p-value = 0.7241
PCS12	3M vs 4 year	Persistent Long Covid	yes	W = 0.97746, p-value = 0.8587
PCS12	3M vs 4 year	Persistent Long Covid	no	W = 0.97642, p-value = 0.3067
PCS12	3M vs 4 year	comorbidities	yes	W = 0.9864, p-value = 0.7425
PCS12	3M vs 4 year	comorbidities	no	W = 0.94664, p-value = 0.3747
PCS12	3M vs 4 year	ICU	yes	W = 0.9133, p-value = 0.4584
PCS12	3M vs 4 year	ICU	no	W = 0.98463, p-value = 0.5284
PCS12	3M vs 4 year	Hospitalized	yes	W = 0.98776, p-value = 0.9652
PCS12	3M vs 4 year	Hospitalized	no	W = 0.96754, p-value = 0.2353
PCS12	3M vs 4 year	Sex	Male	W = 0.9712, p-value = 0.6757
PCS12	3M vs 4 year	Sex	Female	W = 0.98946, p-value = 0.9199
PCS12	3M vs 4 year	readmitted	yes	W = 0.98471, p-value = 0.9792
PCS12	3M vs 4 year	readmitted	no	W = 0.98405, p-value = 0.5061
MCS12	3M vs 4 year	total group		W = 0.97937, p-value = 0.2369
MCS12	3M vs 4 year	Persistent Long Covid	yes	W = 0.98398, p-value = 0.9615
MCS12	3M vs 4 year	Persistent Long Covid	no	W = 0.97016, p-value = 0.1557
MCS12	3M vs 4 year	comorbidities	yes	W = 0.98123, p-value = 0.4827
MCS12	3M vs 4 year	comorbidities	no	W = 0.90704, p-value = 0.0763
MCS12	3M vs 4 year	ICU	yes	W = 0.95263, p-value = 0.7615
MCS12	3M vs 4 year	ICU	no	W = 0.97694, p-value = 0.2081
MCS12	3M vs 4 year	Hospitalized	yes	W = 0.98277, p-value = 0.8652
MCS12	3M vs 4 year	Hospitalized	no	W = 0.97391, p-value = 0.3981
MCS12	3M vs 4 year	Sex	Male	W = 0.97581, p-value = 0.7917
MCS12	3M vs 4 year	Sex	Female	W = 0.97413, p-value = 0.3023
MCS12	3M vs 4 year	readmitted	yes	W = 0.97412, p-value = 0.9264
MCS12	3M vs 4 year	readmitted	no	W = 0.97867, p-value = 0.2685
