# Supplementary material for: Evaluating the longitudinal physical and psychological health effects of persistent long Covid 3.5 years after infection
Source: PLoS One. 2025 Jun 24;20(6):e0326790. doi: 10.1371/journal.pone.0326790 (PMC12186912; doi:10.1371/journal.pone.0326790)
Supplement: S3 Table — (PDF) [file pone.0326790.s004.pdf]

SF12 subscores	time	subgroup	response	shapiro-wilk test
MCS12	1y	comorbidity	no	W = 0.77755, p-value = 0.00773
MCS12	3 month	Comorbidity	no	W = 0.87745, p-value = 0.01939
MCS12	4y	Comorbidity	no	W = 0.88408, p-value = 0.02531
MCS12	1y	comorbidity	yes	W = 0.91521, p-value = 0.002023
MCS12	3 month	Comorbidity	yes	W = 0.9319, p-value = 0.00217
MCS12	4y	Comorbidity	yes	W = 0.89751, p-value = 6.457e-05
MCS12	1y	hospital	no	W = 0.9021, p-value = 0.003374
MCS12	3 month	Hospital	no	W = 0.92875, p-value = 0.006831
MCS12	4y	Hospital	no	W = 0.90827, p-value = 0.001337
MCS12	1y	hospital	yes	W = 0.9151, p-value = 0.06928
MCS12	3 month	Hospital	yes	W = 0.93668, p-value = 0.05442
MCS12	4y	Hospital	yes	W = 0.87738, p-value = 0.000869
MCS12	1y	ICU	no	W = 0.9022, p-value = 0.0003379
MCS12	3 month	ICU	no	W = 0.94321, p-value = 0.002338
MCS12	4y	ICU	no	W = 0.89962, p-value = 1.653e-05
MCS12	1y	ICU	yes	W = 0.90503, p-value = 0.4565
MCS12	3 month	ICU	yes	W = 0.92083, p-value = 0.5114
MCS12	4y	ICU	yes	W = 0.72509, p-value = 0.01124
MCS12	1y	Persistent Long Covid	no	W = 0.87603, p-value = 0.0001574
MCS12	3 month	Persistent Long Covid	no	W = 0.89017, p-value = 5.146e-05
MCS12	4y	Persistent Long Covid	no	W = 0.88011, p-value = 2.024e-05
MCS12	1y	Persistent Long Covid	yes	W = 0.95552, p-value = 0.5818
MCS12	3 month	Persistent Long Covid	yes	W = 0.98062, p-value = 0.8972
MCS12	4y	Persistent Long Covid	yes	W = 0.88265, p-value = 0.007841
MCS12	1y	readmitted	no	W = 0.90428, p-value = 0.0004534
MCS12	3 month	Readmitted	no	W = 0.93668, p-value = 0.001185
MCS12	4y	Readmitted	no	W = 0.89009, p-value = 8.686e-06
MCS12	1y	readmitted	yes	W = 0.94935, p-value = 0.7326
MCS12	3 month	Readmitted	yes	W = 0.90528, p-value = 0.3641
MCS12	4y	Readmitted	yes	W = 0.93786, p-value = 0.5902
MCS12	1y	sex	female	W = 0.90899, p-value = 0.002705
MCS12	3 month	sex	Female	W = 0.93165, p-value = 0.003838
MCS12	4y	sex	Female	W = 0.89973, p-value = 0.000214
MCS12	1y	sex	male	W = 0.88469, p-value = 0.04595
MCS12	3 month	sex	Male	W = 0.94293, p-value = 0.173
MCS12	4y	sex	Male	W = 0.84271, p-value = 0.0008291
PCS12	1y	comorbidity	no	W = 0.62959, p-value = 0.0001248
PCS12	3 month	Comorbidity	no	W = 0.8342, p-value = 0.003751
PCS12	4y	Comorbidity	no	W = 0.73805, p-value = 0.0001639
PCS12	1y	comorbidity	yes	W = 0.95462, p-value = 0.06122
PCS12	3 month	Comorbidity	yes	W = 0.95179, p-value = 0.01756
PCS12	4y	Comorbidity	yes	W = 0.88498, p-value = 2.275e-05
PCS12	1y	hospital	no	W = 0.91022, p-value = 0.005741
PCS12	3 month	Hospital	no	W = 0.87105, p-value = 9.698e-05
PCS12	4y	Hospital	no	W = 0.8394, p-value = 1.376e-05
PCS12	1y	hospital	yes	W = 0.93547, p-value = 0.1771
PCS12	3 month	Hospital	yes	W = 0.982, p-value = 0.8444
PCS12	4y	Hospital	yes	W = 0.88013, p-value = 0.00102
PCS12	1y	ICU	no	W = 0.92865, p-value = 0.003227
PCS12	3 month	ICU	no	W = 0.9261, p-value = 0.0003331
PCS12	4y	ICU	no	W = 0.86618, p-value = 8.758e-07
PCS12	1y	ICU	yes	W = 0.90981, p-value = 0.4814
PCS12	3 month	ICU	yes	W = 0.85544, p-value = 0.1741
PCS12	4y	ICU	yes	W = 0.86926, p-value = 0.2233
PCS12	1y	Persistent Long Covid	no	W = 0.914, p-value = 0.002353
PCS12	3 month	Persistent Long Covid	no	W = 0.91346, p-value = 0.0003768
PCS12	4y	Persistent Long Covid	no	W = 0.83203, p-value = 6.727e-07
PCS12	1y	Persistent Long Covid	yes	W = 0.90319, p-value = 0.0904
PCS12	3 month	Persistent Long Covid	yes	W = 0.92592, p-value = 0.07
PCS12	4y	Persistent Long Covid	yes	W = 0.89307, p-value = 0.013
PCS12	1y	readmitted	no	W = 0.93167, p-value = 0.004709
PCS12	3 month	Readmitted	no	W = 0.91836, p-value = 0.000163
PCS12	4y	Readmitted	no	W = 0.87252, p-value = 1.918e-06
PCS12	1y	readmitted	yes	W = 0.85563, p-value = 0.213
PCS12	3 month	Readmitted	yes	W = 0.84504, p-value = 0.1106
PCS12	4y	Readmitted	yes	W = 0.69556, p-value = 0.002006
PCS12	1y	sex	female	W = 0.90964, p-value = 0.002833
PCS12	3 month	sex	Female	W = 0.93725, p-value = 0.006522
PCS12	4y	sex	Female	W = 0.83398, p-value = 2.066e-06
PCS12	1y	sex	male	W = 0.93179, p-value = 0.2602
PCS12	3 month	sex	Male	W = 0.89236, p-value = 0.01255
PCS12	4y	sex	Male	W = 0.90727, p-value = 0.0197
