# Supplementary material for: Evaluating the longitudinal physical and psychological health effects of persistent long Covid 3.5 years after infection
Source: PLoS One. 2025 Jun 24;20(6):e0326790. doi: 10.1371/journal.pone.0326790 (PMC12186912; doi:10.1371/journal.pone.0326790)
Supplement: S4 Table — A The Fisher’s exact test was used instead of Chi-square test because one of the expected cell values was less than 5 in the statistical comparison. (PDF) [file pone.0326790.s005.pdf]

Comparisons		Timepoints					
		PHQ9(≥5)		GAD7(≥5)		IESR(≥33)	
		3M vs 1Y	3M vs 3.5y	3M vs 1Y	3M vs 3.5y	3M vs 1Y	3M vs 3.5y
all patients		0.570	0.139	0.427	0.064	0.811	0.013
Persistent Long Covid at the 3.5 year timepoint	Persistent Long Covid	0.666[A]	0.141[A]	0.714[A]	0.026[A]	0.701[A]	0.011[A]
	no Long Covid	0.501	0.040	0.560	0.173	0.880	0.213
Hospitalization status at initial infection	hospitalized	0.324	0.209	0.833	0.061	0.763	0.393
	Not hospitalized	0.899	0.390	0.230	0.398	0.544	0.002[A]
Re-admitted at initial infection	Re-admitted to hospital	0.667[A]	0.630[A]	0.673[A]	0.386[A]	1.000[A]	1.000[A]
	not readmitted	0.708	0.211	0.463	0.095	0.811	0.005
Comorbidities present	Comorbidities	0.444	0.087	0.724	0.205	0.680	0.036
	no comorbidities	0.798	0.890	0.231	0.103	1.000[A]	0.155[A]
ICU admission at initial infection	ICU admission	1.000[A]	0.604[A]	1.000[A]	0.103[A]	1.000[A]	N/A
	no ICU	0.615	0.183	0.407	0.156	0.692	0.011
Sex	Male	0.894	0.623	0.435	0.182	0.513	0.222[A]
	Female	0.529	0.142	0.143	0.163	0.448	0.051
