# Supplementary material for: Evaluating the longitudinal physical and psychological health effects of persistent long Covid 3.5 years after infection
Source: PLoS One. 2025 Jun 24;20(6):e0326790. doi: 10.1371/journal.pone.0326790 (PMC12186912; doi:10.1371/journal.pone.0326790)
Supplement: Attachment — Submitted filename: Review Comments_Bala Harri.docx [file pone.0326790.s006.docx]

**Review Comments**

This study is an important and informative contribution to the field, particularly in its investigation of the long-term psychological impact of Long Covid. However, there are few areas that require further clarification and elaboration.

1. **Background and Literature Review:**
   a. While the study aims to assess the mental health impact of Long Covid, including depression, anxiety, and post-traumatic stress disorder (PTSD), the background section does not provide sufficient discussion on the relationship between COVID-19 infection and these mental health outcomes.
   b. The authors should include a review of existing literature that demonstrates how COVID-19 infection, particularly in its early stages, has been linked to depression, anxiety, and PTSD.
   c. Additionally, evidence of the prevalence and severity of these mental health conditions at the time of acute infection, or shortly thereafter, should be incorporated into the background section to contextualize the study’s findings.
2. **Causality of Depression in Long Covid Patients:**
   a. The results indicate that a significantly higher proportion of patients with persistent Long Covid met the criteria for depression at multiple timepoints (3 months, 1 year, and 3.5 years post-infection).
   b. However, the study does not establish COVID-19 as the primary cause of depression. Other potential contributing factors—such as pre-existing mental health conditions, socioeconomic stressors, and pandemic-related disruptions—should be acknowledged in the discussion section.
   c. The authors should also consider discussing whether other confounding factors, such as prolonged physical symptoms, social isolation, or employment difficulties, could have contributed to the persistence of depressive symptoms.
3. **Interpretation of Findings:**
   a. The discussion should clarify whether the persistence of depressive symptoms in Long Covid patients represents a direct consequence of the viral infection, a secondary effect of chronic illness, or an interaction between multiple biopsychosocial factors.
   b. If possible, the authors should compare their findings with other long-term studies on post-viral syndromes to strengthen their conclusions.
4. **Clarity in Reporting:**
   a. The authors should also ensure consistency in terminology when referring to “Long Covid” and clarify how they distinguish persistent Long Covid from other post-infection conditions.

**Conclusion**

Overall, this study provides valuable insights into the long-term psychological health effects of Long Covid. However, addressing the points above—particularly strengthening the background section, discussing alternative explanations for depression would significantly enhance the manuscript’s clarity and scientific rigor.
